# Exogenous Silicon Attenuates Cadmium-Induced Oxidative Stress in *Brassica napus* L. by Modulating AsA-GSH Pathway and Glyoxalase System

**DOI:** 10.3389/fpls.2017.01061

**Published:** 2017-06-19

**Authors:** Mirza Hasanuzzaman, Kamrun Nahar, Taufika Islam Anee, Masayuki Fujita

**Affiliations:** ^1^Department of Agronomy, Faculty of Agriculture, Sher-e-Bangla Agricultural UniversityDhaka, Bangladesh; ^2^Laboratory of Plant Stress Responses, Department of Applied Biological Science, Faculty of Agriculture, Kagawa UniversityTakamatsu, Japan; ^3^Department of Agricultural Botany, Faculty of Agriculture, Sher-e-Bangla Agricultural UniversityDhaka, Bangladesh

**Keywords:** antioxidant defense, heavy metals, plant nutrients, ROS, trace element

## Abstract

Cadmium (Cd) brings a devastating health hazard to human being as a serious consequence of agricultural and environmental contamination. We demonstrated the protective effect of silicon (Si) on cadmium (Cd)-stressed rapeseed (*Brassica napus* L. cv. BINA Sharisha 3) plants through regulation of antioxidant defense and glyoxalase systems. Twelve-day-old seedlings were exposed to Cd stress (0.5 and 1.0 mM CdCl_2_) separately and in combination with Si (SiO_2_, 1.0 mM) for 2 days. Cadmium toxicity was evident by an obvious oxidative stress through sharp increases in H_2_O_2_ content and lipid peroxidation (malondialdehyde, MDA content), and visible sign of superoxide and H_2_O_2_. Cadmium stress also decreased the content of ascorbate (AsA) and glutathione (GSH) as well as their redox pool. The activities of monodehydroascorbate reductase (MDHAR), dehydroascorbate reductase (DHAR) and catalase (CAT) were decreased by Cd while ascorbate peroxidase (APX) and glutathione *S*-transferase (GST) activities were increased. The enzymes of glyoxalase system (glyoxalase I, Gly I and glyoxalase II, Gly II) were also inefficient under Cd stress. However, exogenous application of Si in Cd treated seedlings reduced H_2_O_2_ and MDA contents and improved antioxidant defense mechanism through increasing the AsA and GSH pools and activities of AsA-GSH cycle (APX, MDHAR, DHAR and GR) and glyoxalase system (Gly I and Gly II) enzymes and CAT. Thus Si reduced oxidative damage in plants to make more tolerant under Cd stress through augmentation of different antioxidant components and methylglyoxal detoxification system.

## Introduction

Cadmium (Cd) is one of the most toxic elements of the earth releasing from natural and anthropogenic sources which poses detrimental hazardous effects both in plant and animal kingdoms (Wu et al., 2017). Cadmium exposure interrupts nutrient uptake, inhibits enzyme activities, generates reactive oxygen species (ROS) and damages cell components (Wu J. et al., 2016; Rahman et al., 2017). Cadmium possesses various degrees of phytotoxicity and exhibits potential health problems when accumulated in edible parts of crops (Wu Z. et al., 2016). In plant, Cd threats seed germination and seedling growth (Liu et al., 2012), disrupts photosynthetic machinery (Burzyński and Zurek, 2007) and cellular redox (Wu et al., 2017), damages meristem nucleoli (Qin et al., 2010), and disrupts protein structure (Kabir et al., 2016). Apart from these, Cd-induced growth inhibition, leaf rolling, cholrosis, necrosis, reduced water potential and even death are common phenomena (Sharma and Dubey, 2007; Anjum et al., 2008; Gill and Tuteja, 2011).

Cadmium induces oxidative stress indirectly by enhancing ROS production; such as singlet oxygen (^1^O_2_), superoxide radical (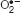), hydrogen peroxide (H_2_O_2_), and hydroxyl radicals (OH^∙^) (Andresen and Küpper, 2013; Rahman et al., 2016). Plants’ antioxidant defense system contains some non-enzymatic antioxidants such as ascorbate (AsA) glutathione (GSH), phenolic compounds, alkaloids, non-protein amino acids, and α-tocopherols as well as a bunch of antioxidant enzymes like catalase (CAT), ascorbate peroxidase (APX), glutathione reductase (GR), monodehydroascorbate reductase (MDHAR), dehydroascorbate reductase (DHAR), and glutathione *S*-transferase (GST) etc. (Hasanuzzaman et al., 2012a; Rahman et al., 2016). The AsA-GSH cycle enzymes are APX, MDHAR, DHAR and GR and a good coordination among these enzymes can also render better tolerance to Cd or any other metal toxicity (Hasanuzzaman et al., 2012b). Highly cytotoxic methylglyoxal (MG) can also be produced in larger amount in plants if exposed to Cd stress. However, the thiol-dependent glyoxalase I (Gly I) and glyoxalase II (Gly II) enzymes can detoxify it by sequential reactions (Rahman et al., 2016; Hasanuzzaman et al., 2017a,b).

Rapeseed (*Brassica napus* L.) is a plant of Brassicaceae family which is grown as oilseed crop, used as leafy vegetable and feed for cattle. Plants of Brassicaceae family are known as metal accumulators having potential roles in phytoextraction (Gall and Rajakaruna, 2013; Ahmad et al., 2015; Mourato et al., 2015). There are several reports demonstrating the performance of *Brassica* spp. as phytoremediator of heavy metal including Cd (Mourato et al., 2015 and references therein). Reduction of oil content and growth performance was reported in *B. juncea* L. under Cd stress (Ahmad et al., 2015). Effect of Cd stress on oxidative stress tolerance and methylglyoxal detoxification system were not studied extensively in rapeseed plant.

Silicon (Si) is considered to be one of the most common elements of the earth crust by mass which has positive roles in diminishing detrimental effects caused by various heavy metals (Greger et al., 2016; Wu J. et al., 2016; Wu Z. et al., 2016; Rahman et al., 2017; Wu et al., 2017). In a recent study, Kabir et al. (2016) reported the Si-mediated mitigation of Cd toxicity in *Medicago sativa* L. by limiting Fe uptake which involves the mechanism of Fe acquisition downregulation. They also noted that Si might have some roles in protecting plants from oxidative stress through modulating antioxidant enzyme activities. In a similar experiment, Wu et al. (2017) also reported Si induced tolerance to oxidative stress where Si reduced the membrane damage modulating the activities of AsA-GSH enzymes. Considering the above facts, the present study has been executed to investigate the role of exogenous Si application in diminishing Cd-induced oxidative stress through regulating AsA-GSH pathway and glyoxalase system in *B. napus* seedlings.

## Materials and Methods

### Plant Materials, Treatments and Design of Experiment

Sterilized uniform seeds of rapeseed (*B. napus* L. cv. BINA Sharisha 3) were grown under controlled conditions (light, 350 μmol photon m^-1^s^-2^; temperature, 25 ± 2°C; relative humidity, 65–70%). Hyponex solution (Hyponex, Japan) was applied as nutrient according to necessity after 5,000-fold dilution (EC 0.849 dS m^-1^; pH 6.0. Twelve-day-old seedlings were treated with 1.0 mM silicon (SiO_2_; Wako, Japan) and 0.5 and 1.0 mM Cd (CdCl_2_; Cadmium Chloride Anhydrous, Wako, Japan). Cadmium concentration of 0.5 and 1.0 mM were considered as mild and severe stress, respectively. Cadmium and Si were applied independently and in combination. The selected dose of Si showed better results under those Cd stresses which were selected after several trial experiments considering oxidative damage or membrane lipid peroxidation level (Supplementary Figure S1) and the phenotypic appearance. Seedlings grown in Hyponex solution only were used as control. Experimental design of this study was completely randomized design (CRD) with three replications. Data were taken after 48 h.

### Measurement of Lipid Peroxidation

Malondialdehyde (MDA) content was estimated to measure the level of lipid peroxidation using thiobarbituric acid (TBA) reagent for extraction of leaves (Heath and Packer, 1968; Hasanuzzaman et al., 2017a).

### Determination of Hydrogen Peroxide Content

Potassium-phosphate (K-P) buffer (pH 6.5) was used for extracting the leaves and centrifugation was done at 11,500 ×*g*. After that, a mixture of titanium tetrachloride (TiCl_4_) and 20% sulphuric acid (H_2_SO_4_) (v/v) was added to the supernatant. The final mixture was read spectrophotometrically at 410 nm (Yu et al., 2003).

### Histochemical Detection of Hydrogen Peroxide and Superoxide

The H_2_O_2_ and 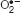 were localized histochemically (Chen et al., 2010) by staining leaves with 1% 3,3-diaminobenzidine (DAB) and 0.1% nitroblue tetrazolium chloride (NBT) solution, respectively.

### Extraction and Measurement of Ascorbate and Glutathione

Measurement of ascorbate and glutathione was done by using leaves homogenized in 5% meta-phosphoric acid containing 1 mM ethylenediaminetetraacetic acid (EDTA) and then centrifuging at 11,500 ×*g* for 12 min at 4°C. The AsA and dehydroascorbate (DHA, oxidized form of AsA) content was assayed following the methods of Huang et al. (2005) and (Nahar et al., 2016a,b). Glutathione and glutathione disulfide (GSSG, oxidized form of GSH) was examined following the method of Yu et al. (2003) and Hasanuzzaman et al. (2017a).

### Determination of Protein

The amount of protein from each sample was determined using bovine serum albumin (BSA) as a protein standard (Bradford, 1976). Different concentrations of solution were prepared with BSA to make standard curve which was used to determine the protein concentration of each plant sample.

### Enzyme Extraction and Assays

Leaf tissue was homogenized in 1 mL of 50 mM ice-cold K-P buffer (pH 7.0) containing 100 mM KCl, 1 mM ascorbate (AsA), 5 mM β-mercaptoethanol, and 10% (w/v) glycerol. The homogenates were centrifuged at 11,500 ×*g* for 10 min, and the supernatants were used to measure enzyme activity (Hasanuzzaman et al., 2017a).

Ascorbate peroxidase (EC: 1.11.1.11) activity was measured according to Nakano and Asada (1981) with a solution mixture of K-P buffer (pH 7.0), AsA, H_2_O_2_, EDTA, and enzyme extract which was read at 290 nm (Hasanuzzaman et al., 2017a).

Monodehydroascorbate reductase (EC: 1.6.5.4) activity was determined following the method described in Hossain et al. (1984). The reaction mixture contained Tris–HCl buffer (pH 7.5), NADPH, AsA, AO, and enzyme solution which was read at 340 nm (Hasanuzzaman et al., 2017a).

Dehydroascorbate reductase (EC: 1.8.5.1) activity was assayed according to the method of Nakano and Asada (1981). The reaction buffer contained K-P buffer (pH 7.0), GSH, EDTA, and dehydroascorbate (DHA), plant sample and it was read at 265 nm (Hasanuzzaman et al., 2017a).

Glutathione reductase (EC: 1.6.4.2) activity was measured according to the method of Hasanuzzaman et al. (2017a) by monitoring absorbance at 340 nm. The reaction mixture contained K-P buffer (pH 7.0), EDTA, GSSG, NADPH, and enzyme extract.

Glutathione *S*-transferase (EC: 2.5.1.18) activity (Hossain et al., 2006): The reaction mixture contained 100 mM Tris–HCl buffer (pH 6.5), 1.5 mM GSH, 1 mM 1-chloro-2,4-dinitrobenzene (CDNB), and enzyme solution which was read at 340 nm (Hasanuzzaman et al., 2017a).

Catalase (EC: 1.11.1.6) activity was determined following the method of Hasanuzzaman et al. (2017a) by monitoring absorbance at 240 nm. Enzyme extract was added with the reaction mixture containing K-P buffer (pH 7.0) and H_2_O_2_.

Gly I (EC: 4.4.1.5) activity was determined following the method of Hasanuzzaman et al. (2017a). The assay mixture consisted of K-P buffer (pH 7.0), MgSO_4_, GSH, MG, and enzyme extract which was read at 240 nm.

Gly II (EC: 3.1.2.6) activity was determined according to Principato et al. (1987) and Hasanuzzaman et al. (2017a). Reaction mixture contained Tris–HCl buffer (pH 7.2), 5,5-dithio-bis (2-nitrobenzoic acid) (DTNB), *S*-D-lactoylglutathione (SLG), enzyme extract, and absorbance was recorded at 412 nm.

### Statistical Analysis

The data were subjected to analysis of variance (ANOVA), and the mean differences were compared by Tukey’s honest significant difference (HSD) test using XLSTAT v.2017 (Addinsoft, 2017). Differences at *P* ≤ 0.05 were considered significant.

## Results

### Oxidative Damage

**FIGURE 1:**
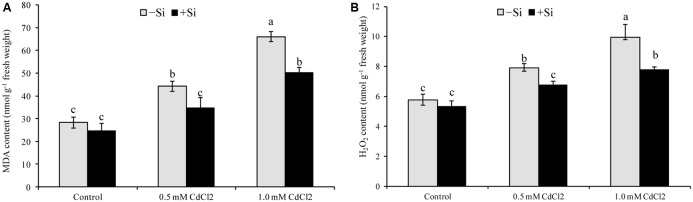
Silicon-induced changes in oxidative stress markers (MDA, **A** and H_2_O_2_, **B** content) in *Brassica napus* seedlings grown under Cd stress. Values (Mean ± SD) of each treatment are obtained from three replications. Bars with different letters are significantly different at *P* < 0.05 applying Tukey’s HSD test.

**FIGURE 2:**
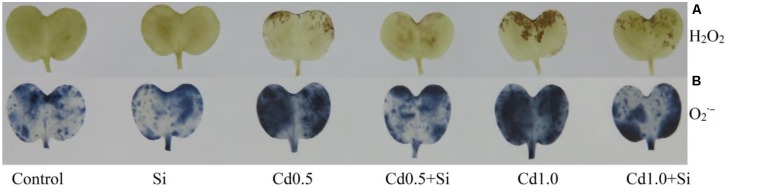
Histochemical detection of H_2_O_2_ **(A)** and 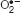
**(B)** in leaves of *Brassica napus* seedlings grown under Cd stress induced by exogenous Si. Si, Cd0.5 and Cd1.0 indicate 1.0 mM SiO_2_, 0.5 mM CdCl_2_ and 1.0 M CdCl_2_, respectively.

Membrane lipid peroxidation increased under Cd stress indicated by increased MDA contents by 56% and 133% in mild and severe stress, respectively, compared with control (**Figure 1A**). Hydrogen peroxide content also rose significantly under Cd stress (**Figure 1B**). However, exogenous Si application reduced both the MDA and H_2_O_2_ contents (**Figure 1**) in Cd-affected seedlings, compared to Cd alone. As an indicator of oxidative stress, H_2_O_2_ and 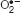 were determined through histochemical staining. Leaves of the Cd-stressed plants showed brown spots of H_2_O_2_ and dark blue spots of 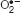 (**Figure 2**) which were prominently evident, compared to control. However, exogenous Si application decreased those spots noticeably from the leaves of Cd affected plants.

### Ascorbate and Glutathione Pool

**FIGURE 3:**
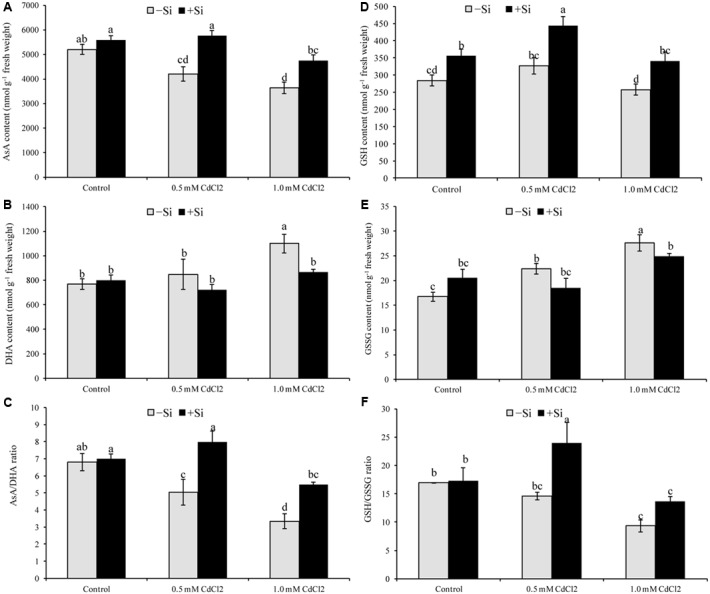
Silicon-induced changes in ascorbate (AsA) **(A)**, glutathione (GSH) **(D)**, DHA **(B)**, GSSG **(E)** and their redox pool **(C,F)** in *Brassica napus* seedlings grown under Cd stress. Values (Mean ± SD) of each treatment are obtained from three replications. Bars with different letters are significantly different at *P* < 0.05 applying Tukey’s HSD test.

Ascorbate content and AsA/DHA ratio decreased under both levels of Cd stress but DHA content enhanced only in case of higher concentration of Cd (**Figures 3A–C**). Glutathione only decreased with higher level of stress, compared to control. Higher GSSG content was recorded in both levels of stress with a reduction in GSH/GSSG ratio (**Figures 3D–F**). Silicon supplementation decreased DHA content but increased AsA content and AsA/DHA ratio; Si addition with Cd decreased GSSG and increased GSH and GSH/GSSG ratio, compared to the Cd stress alone (**Figures 3A–F**).

### Activities of Antioxidant Enzymes

#### AsA-GSH Cycle Enzymes

**FIGURE 4:**
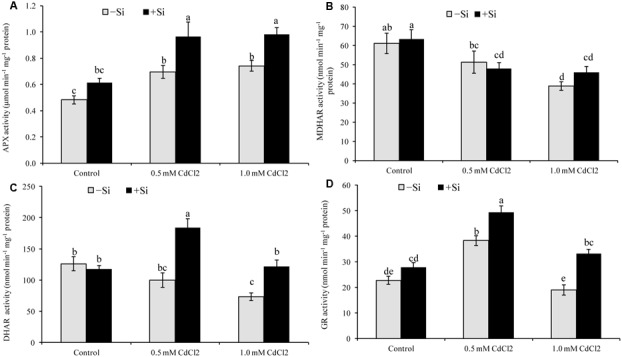
Silicon-induced changes in the activities of AsA-GSH cycle enzymes (APX, MDHAR, DHAR and GR presented in **A–D**, respectively) in *Brassica napus* seedlings grown under Cd stress. Values (Mean ± SD) of each treatment are obtained from three replications. Bars with different letters are significantly different at *P* < 0.05 applying Tukey’s HSD test.

The activity of APX increased by 43 and 53% under mild and severe stress, respectively, compared to control. Si supplementation with Cd further increased its activity (**Figure 4A**). Cadmium stress reduced the activities of MDHAR and DHAR in both levels of stress. After Si application, activity of DHAR increased by 84 and 66% in mild and severe stresses, respectively, compared to the non-treated stressed seedlings (**Figures 4B,C**). Comparing with control, GR activity increased and decreased under mild and severe Cd stress, respectively. Silicon addition increased GR activity by 29 and 75% in mild and severe stress, respectively, compared to stress alone(**Figure 4D**).

#### Other Antioxidant Enzymes

**FIGURE 5:**
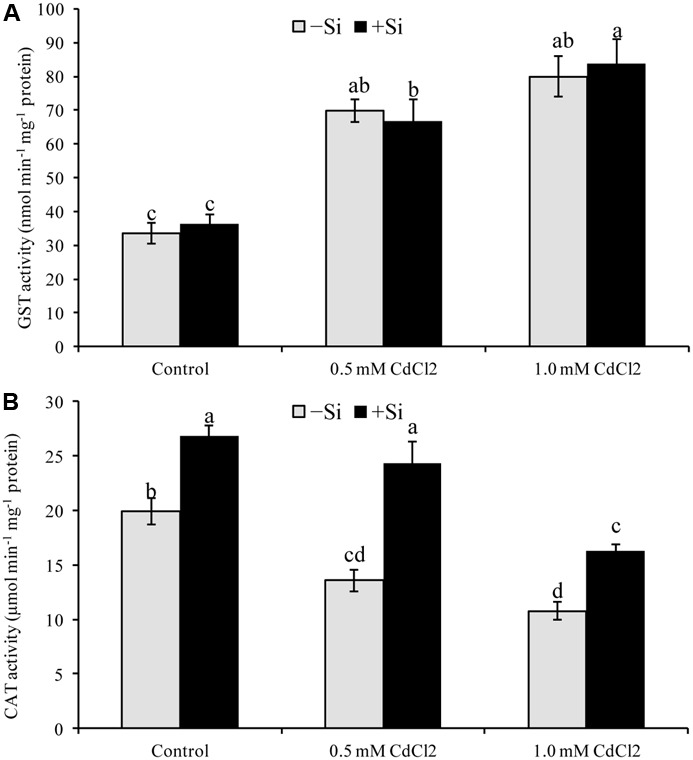
Silicon-induced changes in the activities of glutathione *S*-transferase (GST) **(A)** and catalase (CAT) **(B)** in *Brassica napus* seedlings grown under Cd stress. Values (Mean ± SD) of each treatment are obtained from three replications. Bars with different letters are significantly different at *P* < 0.05 applying Tukey’s HSD test.

Seedlings exposed to Cd stress reduced CAT activity, compared to control. In contrast, Si addition increased CAT activity by 79 and by 51% for mild and severe stress, respectively, compared to Cd stress alone (**Figure 5B**). Though GST activity upregulated by 108 and 139% under mild and severe stress, respectively (compared with control), Si supplementation didn’t change its activity further (**Figure 5A**).

### Glyoxalse System Enzymes

**FIGURE 6:**
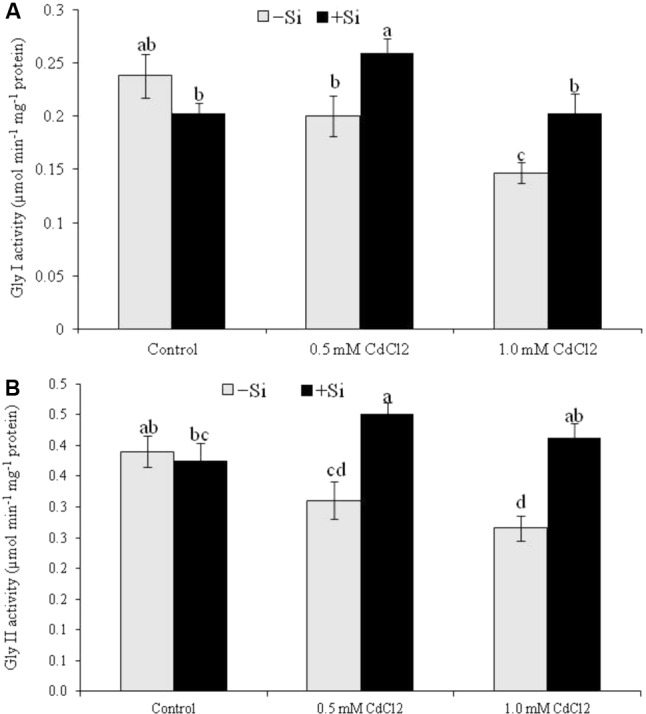
Silicon-induced changes in the activities of glyoxalase enzymes (Gly I and Gly II activity presented in **A** and **B**, respectively) in *Brassica napus* seedlings grown under Cd stress. Values (Mean ± SD) of each treatment are obtained from three replications. Bars with different letters are significantly different at *P* < 0.05 applying Tukey’s HSD test.

Rapeseed seedlings exposed to Cd stress reduced Gly I activity by 16% under mild stress and by 38% under severe stress, compared to control. Cadmium stress also reduced Gly II activity (by 20 and 32% under mild and severe stress, respectively). Silicon addition with Cd increased both Gly I and Gly II activities under both levels of stress (compared to Cd stress alone) (**Figures 6A,B**).

### Phenotypic Appearance of Seedlings

**FIGURE 7:**
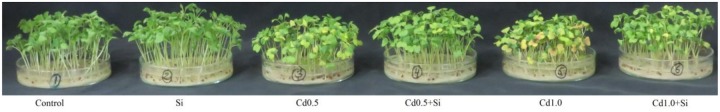
Phenotypic appearance of *Brassica napus* seedlings grown under Cd stress induced by exogenous Si. Si, Cd0.5 and Cd1.0 indicate 1.0 mM SiO_2_, 0.5 mM CdCl_2_ and 1.0 M CdCl_2_, respectively.

Cadmium stress resulted in chlorosis/leaf yellowing symptom, Cd stress also decreased seedlings vigor, compared to control seedlings. Exogenous Si improved phenotypic appearance of seedlings improving the seedlings vigor and alleviating chlorosis symptom (compared to Cd stressed seedlings without Si) (**Figure 7**).

## Discussion

Cadmium does not participate in Fenton reaction (Clemens, 2006). Cd amplifies free Fe^++^ ion by displacing it from active sites which enhances Fenton reaction and ROS production (Romero-Puertas et al., 2004). Cadmium indirectly activates NADPH oxidase activity (Romero-Puertas et al., 2004), impairs stomatal movement, photosynthetic machinery (Islam et al., 2008), CO_2_ fixation enzymes (Romero-Puertas et al., 2004) and enhances ROS production. In this study, the *B. napus* seedlings treated with Cd showed oxidative damage (increased H_2_O_2_ production and MDA content) corroborating the results of previous studies (Andresen and Küpper, 2013; Srivastava et al., 2015). Leaves of the Cd-stressed plants showed brown spots of H_2_O_2_ and dark blue spots of 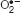. An identical pattern of oxidative stress and damage was noticed in Cd affected mung bean seedlings (Nahar et al., 2016b).

The addition of Si in Cd-treated rapeseed plants reduced the spots of H_2_O_2_ and 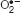, decreased H_2_O_2_ and lipid peroxidation/MDA level enhancing the antioxidant defense mechanism (compared to Cd treated plant only). The presence of Si in plant growing medium decreases Cd uptake through root and then decreases the transfer of Cd to shoot which reduces Cd-induced cellular damages (Srivastava et al., 2015; Tang et al., 2015). Decreasing Cd uptake and increasing antioxidant enzymes and photosynthesis Si reduced oxidative stress in cotton plant (Farooq et al., 2013). Results of several other studies are also supportive of the investigation of the present study (Srivastava et al., 2015; Tang et al., 2015).

Ascorbate is potent water soluble ROS scavenger of cell converting H_2_O_2_ to H_2_O by the activity of APX (Hasanuzzaman et al., 2012a; Gill et al., 2015). Ascorbate content and AsA/DHA ratio declined, APX activity increased under Cd stress which was accountable for increasing H_2_O_2_ level (Hasanuzzaman et al., 2017a). The enzymes MDHAR and DHAR take part in the regeneration of AsA from its oxidative state DHA (Hasanuzzaman et al., 2012a). So, a decrease of AsA content in Cd affected seedlings of this study is corroborating with the decrease activities of MDHAR and DHAR. But Si addition with Cd increased the activities of MDHAR and DHAR, and AsA restoration, AsA/DHA ratio decreasing DHA content (compared to Cd stress alone). When Si was supplemented with Cd treatments the seedlings also showed higher APX activity, compared to Cd treatments alone which is supported by the findings of other studies (Tang et al., 2015). Enhanced activities of AsA-GSH cycle enzymes APX, MDHAR, GR with enhanced levels of AsA and GSH were induced by exogenous Si application in Chilling stressed cucumber leaves which alleviated the oxidative stress (Jiao-jing et al., 2009).

Glutathione having vital biological functions is a water soluble antioxidant of non-protein thiol group, profusely dispersed in the cytosol, chloroplast, cytoplasm, apoplast, mitochondria, and peroxisome. It scavenges a range of ROS viz., H_2_O_2_, OH^∙^, and ^1^O_2_ (Anjum et al., 2012; Hasanuzzaman et al., 2012a; Gill et al., 2013). In the present study, GSH level did not change but the GSSG level increased highly under Cd stress that resulted in a reduced GSH/GSSG ratio, compared to control. Glutathione reductase catalyzes the reaction involved in transformation of GSSG to GSH. Under mild Cd stress the activity of GR increased but that was not enough to restore and increase GSH content significantly. Under severe Cd stress, both GR activity and GSH level diminished. Similar trend of GSH and GSSG pool, and GR activity were reported previously under Cd stress (Hasanuzzaman et al., 2017a). When Si was co-applied with Cd the activity of GR increased in rapeseed seedlings which renovated and augmented content of GSH, dropped off GSSG level to increase the GSH/GSSG ratio which is comparable with previous studies (Srivastava et al., 2015; Tang et al., 2015).

Catalase presenting in different cell organelles (Garg and Manchanda, 2009) boosts up ROS scavenging process with its highest capacity to scavenge upto six million H_2_O_2_ in a minute (Gill and Tuteja, 2010). The activity of CAT decreased noticeably due to Cd exposure which is substantiating with the increased H_2_O_2_ level, compared to control. Exogenous Si supplementation restored and augmented CAT activity of Cd affected rapeseed seedlings which decreased H_2_O_2_ generation, compared to Cd treatment only which is supported by a similar previous study with rice (Srivastava et al., 2015).

Glutathione *S*-transferases presenting in apoplast, cytosol, chloroplast, mitochondria catalyze the conjugation of xenobiotic substrates and GSH. The activities of GSTs were found to be upregulated in plants under Cd and other stresses as well (Dixon et al., 2010; Hasanuzzaman et al., 2012b, 2017a) that support increased GST activity of Cd affected seedlings of our study. The activity of GST did not increase further in Cd affected seedlings supplemented with exogenous Si. But Debona et al. (2014) demonstrated Si induced enhancement of GST activity in wheat leaves.

Methylglyoxal is an α-oxoaldehyde, highly reactive and cytotoxic compound production of which is spontaneous via different enzymatic and non-enzymatic reactions. Methylglyoxal is amplified 2- to 6-fold under stress condition (than the control) and with its cytotoxic capacity MG damages ultrastructural cellular components including DNA and can cause mutation (Yadav et al., 2005; Hasanuzzaman et al., 2017b). Glyoxalase system poses glyoxalase I (Gly I) and glyoxalase II (Gly II) enzymes which utilize GSH as co-factor to detoxify MG (Hasanuzzaman et al., 2017b). Cadmium stress decreased Gly I and Gly II activity, compared to the control treatment indicating brake down MG detoxification system by Cd toxicity. Mung bean plants (Nahar et al., 2016a) and rapeseed plants (Hasanuzzaman et al., 2017a) demonstrated similar pattern of response of glyoxalase system enzymes under Cd stress. Treatment with Si improved the activities of Gly I and Gly II and also the content of GSH indicating the crucial roles of Si in MG detoxification under Cd stress.

The results reveal that Si alleviated oxidative stress as it decreased H_2_O_2_ content and membrane lipid peroxidation. The mechanism was Si enhanced components of antioxidant defense system which decreased oxidative stress. Among the studied antioxidant components, Si significantly upregulated AsA and GSH levels, increased activities of APX, DHAR, GR and CAT those scavenged ROS and decreased oxidative damage. The reduction of oxidative damage was also imparted by Si-induced improved glyoxalase system which decreases MG generation and subsequent oxidative damage. The overall advantageous effect of Si was reflected in phenotypic appearance of Cd affected rapeseed seedlings where Si supplementation alleviated chlorosis and improved seedlings vigor.

## Conclusion

Our results suggest that exogenous Si serves well in regulating antioxidant metabolism in *B. napus* seedlings under Cd stress. Silicon-mediated coordinated actions of AsA-GSH pathway and glyoxalase systems maintained the redox state of AsA and GSH and minimized the Cd-induced oxidative damages. This also indicates a central role of GSH because of its relations with both antioxidant defense systems and glyoxalase systems. The signaling roles of Si in regulating biosynthesis of metabolites and regulation of stress-induced genes and their relation to preventing stress affects and contributing stress tolerances need further inspection. Further research should be focussed on the interrelation of Si with other signaling molecules such as nitric oxide (NO), polyamines and phytohormones.

## Author Contributions

MH and MF conceived and designed the experiments. MH and KN performed the experiments. MH and TA analyzed the data. MF contributed reagents/materials/analysis tools. MH, KN, and TA wrote the manuscript. All authors read and approved the final manuscript.

## Conflict of Interest Statement

The authors declare that the research was conducted in the absence of any commercial or financial relationships that could be construed as a potential conflict of interest.
