# Supplementary material for: Exogenous Silicon Attenuates Cadmium-Induced Oxidative Stress in Brassica napus L. by Modulating AsA-GSH Pathway and Glyoxalase System
Source: Front Plant Sci. 2017 Jun 19;8:1061. doi: 10.3389/fpls.2017.01061 (PMC5475239; doi:10.3389/fpls.2017.01061)
Supplement: Supplementary file 1 [file Image_1.PDF]

Exogenous silicon attenuates cadmium-induced oxidative stress in *Brassica napus* L. by modulating AsA-GSH pathway and glyoxalase system

Mirza Hasanuzzaman[1*], Kamrun Nahar[2,3], Taufika Islam Anee[1,2] and Masayuki Fujita[2]
[1]*Department of Agronomy, Faculty of Agriculture, Sher-e-Bangla Agricultural University, Sher-e-Bangla Nagar, Dhaka 1207, Bangladesh*
[2]*Laboratory of Plant Stress Responses, Department of Applied Biological Science, Faculty of Agriculture, Kagawa University, Miki-cho, Kita-gun, Kagawa 761-0795, Japan*
[3]*Department of Agricultural Botany, Faculty of Agriculture, Sher-e-Bangla Agricultural University, Sher-e-Bangla Nagar, Dhaka 1207, Bangladesh*

[*]Corresponding author: Mirza Hasanuzzaman; e-mail: mhzsauag@yahoo.com; Tel: +8801716587711

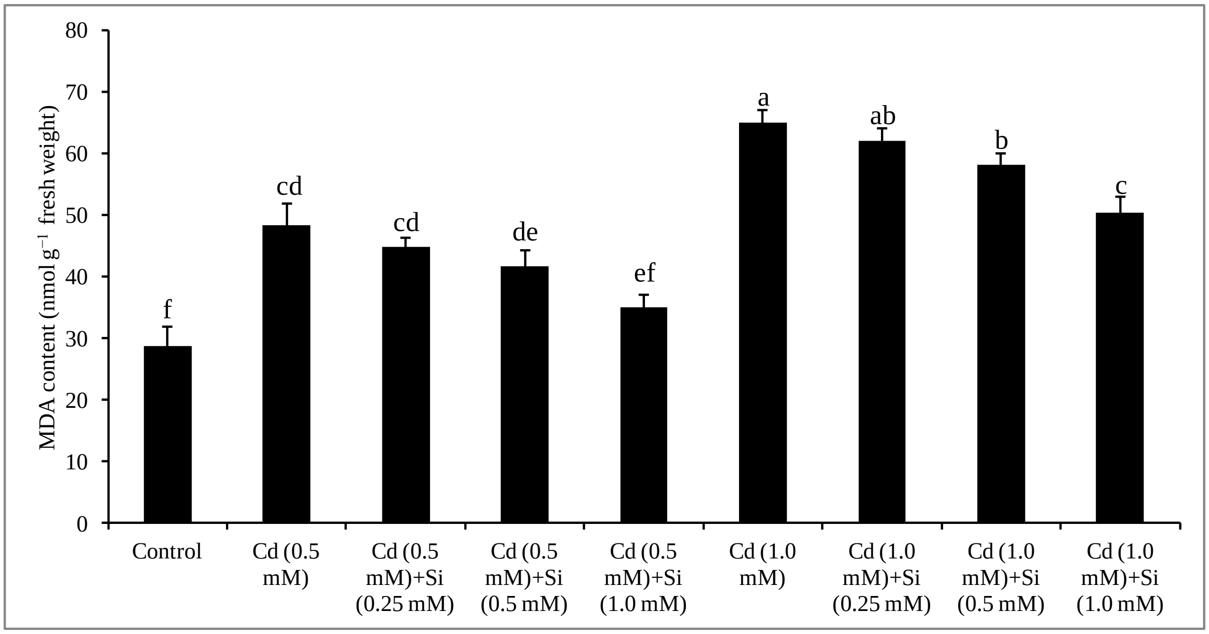

Fig. S1 MDA (malondealdehyde, a product of lipid peroxidation) content in *Brassica napus* seedlings induced by different levels of cadmium (Cd) and silicon (Si). Different doses of Si (0.25 mM, 0.5 mM and 1.0 mM $CdCl_2$) and different doses of Cd (0.5 and 1.0 mM $CdCl_2$) were applied separately and in combined to observe the effect of Si in Cd affected seedlings. Values (Mean±SD) of each treatment are obtained from three replications. Bars with different letters are significantly different at $P < 0.05$ applying Tukey's HSD test.
